# Ribonucleases of Mice and Men: Unveiling the Roles of the RNase A Superfamily in Host Defence

**DOI:** 10.1155/jimr/3940139

**Published:** 2025-11-10

**Authors:** Raúl Anguita, Yusuf Ali, Brian Becknell, Ester Boix

**Affiliations:** ^1^ Department of Biochemistry and Molecular Biology, Faculty of Biosciences, Universitat Autònoma de Barcelona (UAB), Cerdanyola del Vallès, 08193, Spain, uab.cat; ^2^ Kidney and Urinary Tract Center, The Abigail Wexner Research Institute at Nationwide Children’s Hospital, Columbus, 43205, Ohio, USA

**Keywords:** antimicrobial proteins, host defence proteins, infectious diseases, mouse model, RNases

## Abstract

Antimicrobial peptides and proteins (AMPs) constitute ancient host defence mechanisms to preserve tissue sterility and protect the host from infectious diseases. Currently, AMPs are awakening the interest of medical researchers due to their potential to become novel weapons to target multi‐drug resistant (MDR) pathogens and thereby overcome the limitations of traditional antibiotics. Among AMPs, human RNases belonging to the RNase A superfamily stand out as promising agents for therapeutic uses given their high antimicrobial activity, wide spectrum against multiple pathogens and low toxicity. However, a better understanding of how human RNases perform their antimicrobial actions in tissues is necessary to develop novel therapies. Mouse infectious disease models can be extremely useful to study the function of AMPs in vivo and have already provided valuable knowledge about RNase role in tissues such as the intestine and urinary tract. Therefore, it is necessary to understand the genetic and functional divergences that exist between human and mouse RNases to design experiments that are poised for clinical translation. The aim of this review is to present the similarities and differences between human and mouse RNases at genomic, structural and functional levels as a guide for future scientists exploring the roles of RNases in host defence.

## 1. Introduction

The RNase A superfamily is composed of a group of homologous proteins with RNA hydrolysing activity that are exclusively found in vertebrates [1]. All family members present some common structural traits: a molecular weight in the range of 13–15 kilodaltons, kidney shape configured by alpha and beta structures stabilised by three to four disulphide bridges, a conserved CKXXNTF motif and the catalytic triad composed of two histidine and one lysine residues [2]. Besides, RNase A family are secretory proteins with a common N‐terminal signal peptide. Apart from the shared enzymatic action of RNases, the proteins contribute to maintain host homeostasis by other biological activities [3], such as neo‐vascularisation [4, 5], tissue repair [6, 7], immune system stimulation [8–10] and antimicrobial properties [11–14]. Exhaustive evolutionary studies suggest that the family emerged with host defence tasks given the rapid gene birth‐and‐death and gene sorting processes that occurred during vertebrate evolution [1, 15]. This fast evolutionary divergence is a common feature of other gene families within the immune system [1, 15, 16]. Certain RNases exhibit a broad antimicrobial spectrum, with reported activities against almost all existing pathogen types. The elevated cationicity, due to the overabundance of arginine/lysine residues and the presence of hydrophobic patches, confers to RNases the amphipathic and membrane destabilising characteristics typical of antimicrobial proteins, required to damage pathogen membranes and induce cell death [17]. RNases also exert antiviral activity, mostly against single‐stranded (ss)RNA viruses in a manner that is dependent on catalytic activity [18].

**Figure 2:**
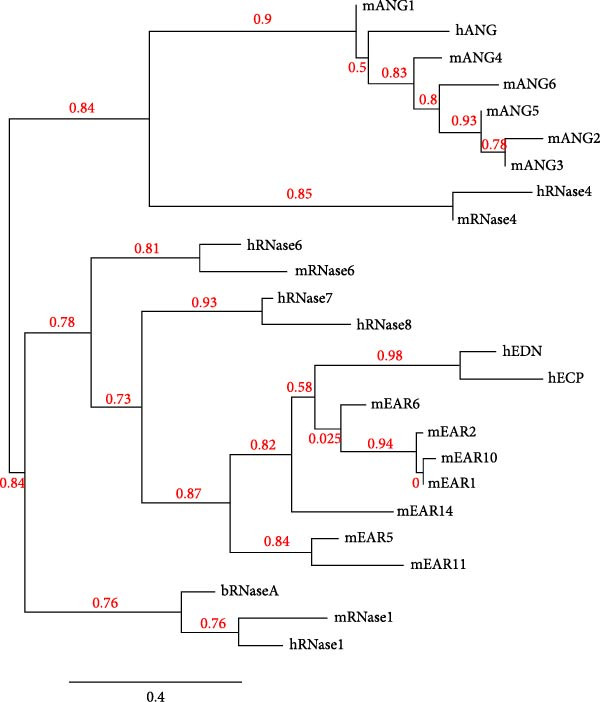
Phylogenetic tree of functional genes of human and mouse RNases. The tree was obtained using phylogeny.fr [25] (https://www.phylogeny.fr/). hEDN, hECP and hANG are also known as RNase 2, RNase 3 and RNase 5, respectively. The phylogenetic tree was constructed by using maximum‐likelihood (ML) method. The scale bar refers to a phylogenetic distance of 0.4 amino acid substitutions per site. The numbers in red indicate the approximate likelihood ratio (aLRT) for the respective branches [26].

While the antimicrobial properties of human RNases have been well described in vitro, the roles of those proteins in vivo are largely unexplored. There are many questions to unravel, such as the specific roles of RNases during the time course of an infection, along with their potential ability to discriminate between commensal and pathogenic microbes. In this context, mouse models are highly valuable experimental tools to simulate infections caused by human pathogens within the context of a mammalian host. Besides, generation of transgenic mice expressing human (h)RNases or containing loss of function mutations for mouse (m)RNases is a powerful strategy to study the expression pattern and role of RNases together with other antimicrobial peptides and proteins (AMPs) during infection [19]. However, the strong evolutionary divergence observed in the RNase A family can be an obstacle when trying to translate functional evidence from mouse to human. While some RNases are structurally and functionally well conserved, with similar properties both in human and mouse, other RNases have experienced rapid divergence and expansion through gene duplication and selection events in rodents [1, 20, 21]. The following sections offer a detailed comparison between human and mouse RNases in the context of infectious diseases and survey evidence obtained in mouse experimental models that are potentially applicable to humans. The review focuses on the eight canonical RNases within the RNase A family. We do not include information on non‐canonical RNases (hRNases 9‐13 and mRNases 9‐12) due to their lack of catalytic activity, though studies are available in mice that suggest their role in regulating male fertility [22]. For better clarity, we have grouped mouse RNases according to each human canonical RNase type. A sequence alignment in Figure 1 illustrates the similarities within each subgroup type, and Figure 2 includes a phylogenetic tree to represent their evolutionary relationship. Complementarily, we have summarised all available information on significant expression changes observed in human and mouse RNase genes during infection, as we seek to advance our understanding of the roles carried out by each corresponding protein in response to infection. Next, we detail currently available information on mouse RNases homologous to each human RNase type.

Figure 1Human and mouse RNase alignment. The RNases are classified in each alignment according to evolutionary proximity, resulting in five distinct groups: (A) pancreatic RNases (RNase1 type, including the bovine RNase A), (B) the eosinophil‐associated RNases, EARs (RNase2 and RNase3 types), (C) RNase 4, (D) angiogenins, ANG (RNase5 type) and (E) RNase 6 and its closest homologues (RNases 6–8 types). Fully conserved residues are highlighted in red, while residues not conserved but having similar properties are marked with red letters. The corresponding secondary structure for each human RNase homologue is indicated above each alignment. Strict β‐turns are denoted by the letters TT, while residues with alternative conformations are marked with a grey asterisk. The disulphide bridges are indicated by green numbers below the alignments. The sequences included are strictly based on transcriptional products and proteins from revised genes according to the Gene Database of the National Institute of Health (NIH). All sequences correspond to gene products from the C57BL/6J reference genome, with the exception of mANG3, which comes from the BALB/c strain genome. Gene IDs from NIH that correspond to the proteins included in the alignment are 282340 (bRNase1/RNase A), 6035 (hRNase 1), 19752 (mRNase 1), 6036 (hRNase2/EDN), 6037 (hRNase3/ECP), 13586 (mEAR1), 13587 (mEAR2), 54159 (mEAR5), 93719 (mEAR6), 93725 (mEAR10), 93726 (mEAR11), 503847 (mEAR14), 6038 (hRNase 4), 58809 (mRNase 4), 283 (hANG), 11727 (mANG1), 11731 (mANG2), 11730 (mANG3) 219033 (mANG4), 503844 (mANG5), 630952 (mANG6), 6039 (hRNase 6), 78416 (mRNase 6), 84659 (hRNase 7) and 122665 (hRNase 8). Mature sequences are included after prediction of signal peptide cleavage using SignalP 5.0 (https://services.healthtech.dtu.dk/services/SignalP-5.0/). The alignment was obtained using *Clustal Omega* [23] (https://www.ebi.ac.uk/jdispatcher/msa/clustalo) while the image was obtained with *ESPript3* [24] (https://espript.ibcp.fr/ESPript/ESPript/).

**Figure:**
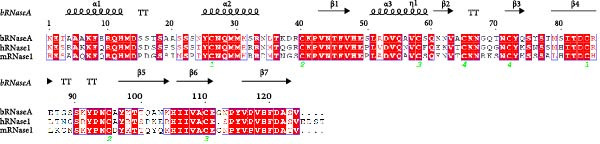
(A)

**Figure:**
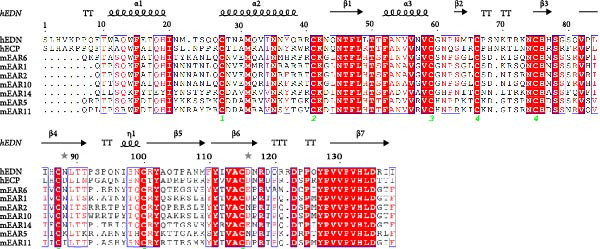
(B)

**Figure:**
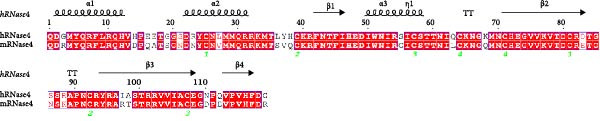
(C)

**Figure:**
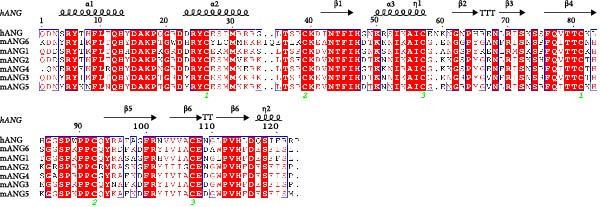
(D)

**Figure:**
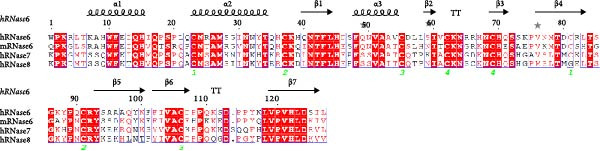
(E)

### 1.1. RNase 1

Among all RNase superfamily members, RNase 1 is the one with the highest enzymatic capacity. Despite sharing 69% identity, some biochemical differences exist between human and mouse RNase 1. First, human RNase 1 has evolved to have a higher optimal pH (7.3) than its mouse homologue (6.4). Second, substrate specificity differs. For example, human RNase demonstrated to be more active against dsRNA than mouse RNase 1. However, these results should be interpreted with caution, as all enzymatic assays were conducted at pH 7.3, the optimal pH for human RNase 1 [27]. In both humans and mice, the major source of RNase 1 in plasma are the vascular endothelial cells, with concentrations in blood and serum of 0.5 μg/mL [28–30]. A principal function of RNase 1 is degradation of extracellular RNA. Accordingly, RNase1^−/−^ mice exhibit higher circulating levels of extracellular RNA, leading to more rapid plasma clotting than wild‐type controls, due to activation of the FXII and FXI coagulation factors [31]. In the context of elevated extracellular RNA in plasma, such as during injury or infection, RNase 1 may be critical to reduce inflammation and predisposition to thrombosis [31]. No antimicrobial activities are generally attributed to RNase 1 [32]. However, activities for hRNase 1 against *Candida albicans*, some *Escherichia coli* strains (25922 and SYY89 but not DR115) and human immunodeficiency virus (HIV) were reported [33, 34].

### 1.2. Eosinophil‐Associated RNases (EARs)

**Figure 3:**
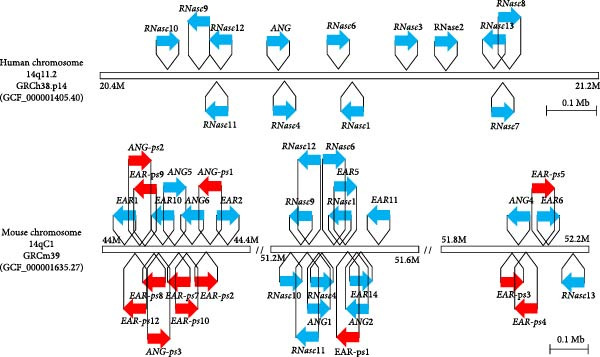
Chromosomal locations of human and mouse RNases genes. Chromosomal positions of genes are drawn in scale based on the reference genomes GRCh38 and GRCm39 (C57BL/6J strain). Functional genes are represented by blue arrows while pseudogenes are represented by red arrows. Official gene names according to NIH datable are included, with *RNASE2* and *RNASE3* genes encoding EDN and ECP, respectively, and *ANG* encoding RNase 5.

The EARs constitute an RNase A subfamily whose members are mainly found in eosinophil granules and are released in response to degranulation. Two EARs are present in human: RNase 2, also called eosinophil derived neurotoxin (EDN), and RNase 3, called eosinophil cationic peptide (ECP). EARs form a unique evolutionary cluster in mammals, with a higher identity percentage in comparison to any other RNase sequence. This RNase subfamily has expanded to multiple genes and pseudogenes in rodents (Figure 3).

Despite the diversity of mouse EARs (mEARs), it is unclear whether all mEARs are actively transcribed genes [1]. Different evolutionary processes with independent gene duplication events have occurred since the divergence between primate and rodents EARs. In primates, EDN and ECP diverged relatively recently with ECP acquiring non‐silent mutations at a higher rate under a strong Darwinian selection. Despite this fast evolution of ECP with respect to EDN, the two sequences are close to 70% identical and share an approximate 50% identity with mEARs [35]. The duplication events responsible for the formation of mEARs occurred during three unique episodes during the past 18 million years [36]. The first event determined the divergence of mEAR5 and 11 from the rest of proteins (Figure 2), and the other two events are responsible for the heterogeneity among the other members. This dynamic birth‐and‐death process enabling the mEAR subfamily formation is shared between proteins whose function is related to host defence, such as major histocompatibility complex (MHC), immunoglobulins (Igs) and T‐cell receptor (TCR), and may enable the specialisation of those proteins to target individual pathogenic species [37, 38]. All human and mouse EARs conserve the catalytic centre and the characteristic four disulphide bridges. However, some unique motifs are present in mEARs with respect to human EARs, such as a four residue deletion near the amino terminal end of the protein [36].

Many similarities can be found between the expression patterns of human and mouse EARs. While eosinophils are the main cellular type that produce EARs, in both human [39, 40] and mice [41] species, macrophages and neutrophils have been identified as additional sources [42–44]. Accordingly, other common tissues with high transcript levels of EARs are bone marrow, spleen and thymus [36, 45]. Expression of EDN, in human, and mEAR6, in mice, can also be detected in liver [45–47]. Moreover, EDN, mEAR2 and mEAR11 have been detected in the human or mouse lung [42, 45, 48]. Expression under homeostatic conditions cannot be detected for all EARs. In human samples, EDN transcripts were detected in the abovementioned tissues at baseline in the absence of inflammatory stimulus, but ECP was not. Similarly, under homeostatic conditions in mice only mEAR1 and mEAR2 are expressed [49]. Therefore, some EARs require an inflammatory stimulus to be expressed [42, 47, 50–54]. In both human and mouse eosinophils, degranulation is activated in response to infection [50, 51, 55] or allergic response [42, 56], enabling the release of EARs to the extracellular space. However, eosinophil degranulation in mouse lungs is less common than in humans and is therefore not a prevalent feature of allergen‐induced responses and asthma in established mouse models [57, 58]. Given that mice are obligatory nasal breathers, those differences between human and mouse eosinophil degranulation may be attributed to diminished presence of inflammatory signals in mouse lungs due to increased nasal air filtration. Besides, human and mouse eosinophils may occupy different local immune microenvironments in the lung and might exert slight distinct roles in inflammatory and tissue remodelling processes [59]. The mechanisms that activate eosinophil degranulation are very similar in human and mouse species. The CCR3‐mediated eosinophil secretion pathway, which is activated through CCL11 (also known as eotaxin‐1), is shared between humans and mice and requires the activation of cell‐signalling factors such as Gi protein α subunit (Giα), phosphatidylinositol 3‐kinase (PI3K), extracellular regulated kinase (ERK) and p38 mitogen‐activated protein kinase (p38 MAPK). Cytokine‐mediated EAR secretion also requires β1 and β2 integrins [41]. Besides, isolated eosinophil‐free granules can still respond to stimuli and secrete EARs both in mice and human [60].

The principal roles attributed to EARs are related to host defence responses. In humans, EDN and ECP antimicrobial activities are clearly distinct from one another. EDN host‐defence function is mostly related to antiviral responses, showing activity against multiple viruses such as respiratory syncytial virus (RSV) [61], HIV [34, 62], hepatitis B virus (HBV) [63] and parainfluenza virus (PIV) [64]. In contrast, amino acid substitutions incorporated during the fast evolutionary process of ECP generated a protein with strong anti‐bacterial [12], anti‐fungal [65] and anti‐parasitic activities [66]. These amino acid substitutions are responsible for the increased isoelectric point (pI) of ECP (10.8) with respect to EDN (8.7) [16]. Interestingly, the cytotoxic capacities of ECP can be replicated in EDN through the addition of some amino acid substitutions at a few key surface residues that interact with pathogen membranes or trigger protein aggregation and cell agglutination [67, 68].

Many antimicrobial properties have also been described for mEARs. Recombinant mEARs 5, 7 and 11 demonstrated equivalent or even stronger in vitro activities than ECP against *E. coli*, *Bacillus subtilis* and *Leishmania donovani*. Surprisingly, mEAR 2 demonstrated little activity against the abovementioned organisms despite being the most toxic against several human cell lines [21]. Those differences in cytotoxicity and antimicrobial activities between mEARs may relate to the high variation of pI (8.2–9.8) [36, 69] and structural variations affecting cationic clusters or hydrophobic patches [17]. *L. donovani* is not the only parasite that is susceptible to EAR antimicrobial activity. A role in antiparasitic action by ECP is observed in vitro [66, 70–79], which is in line with multiple studies correlating increased ECP levels with parasitic infection [52, 54, 80–85]. Besides, the prevalence of the 434(G > C) polymorphism of ECP, which results in an arginine to threonine substitution in the 97 position of the mature protein, in *Schistosoma mansoni* endemic areas of Africa indicates the potential role of this protein in combating parasitic infections [86]. In mice, hepatic expression of mEARs 1, 2 and 6 is induced during *S. mansoni* infection. While mEARs 1 and 2 can be detected in liver during homeostatic conditions and their elevated levels during *S. mansoni* infection clearly correlated with eosinophilia, mEAR 6 expression is specifically induced in liver in response to the parasite [47]. Further experiments are required to unveil the potential roles that mEARs may play in mouse immune responses against *S. mansoni* and other parasites.

Many studies have also described antiviral activities for mEARs, especially against respiratory viruses. Expression of mEARs 1 and 2 is induced in response to RSV infection, and hypereosinophilic IL‐5 transgenic mice demonstrate increased mEar levels and accelerated viral clearance in the lung [50]. However, loss‐of‐function experiments have not been performed in vitro or in vivo to attribute increased viral clearance specifically to mEARs [50]. During the infection by the pneumonia virus of mice (PVM), which is related to RSV, eosinophil infiltration occurs initially in lungs and correlates with improved recovery [87, 88]. Besides, mEAR 11 expression in lung is induced in IFN‐αβR^−/−^ mice with respect to wild‐type mice during PVM infection and may be a key factor in sustaining the antiviral response in an otherwise lethal pneumonia [51]. In line with this evidence, an in vitro study revealed that recombinant mEAR2 causes a six‐fold reduction in PVM infectivity in respiratory epithelial cells in vitro [48]. Interestingly, during PVM strain J3666 infection, mEAR2 expression was downregulated in mice lungs while mEAR1 expression was induced. In contrast, no mEAR2 downregulation was detected when infecting mice with PVM strain 15, and the authors suggested that strain J3666 may possess evasion mechanisms for the host’s antiviral response [48]. Another respiratory virus to which mEARs confers protection during infection is influenza A virus. Premature children are at risk of suffering bronchopulmonary dysplasia (BPD) and are more susceptible to viral respiratory infections. In a mouse model of BPD, mEAR1 expression was found to be downregulated. The critical role of mEAR1 in host defence was demonstrated as electroporation‐mediated *Ear1* gene delivery to the lung recovered the epithelial expression of mEAR1 and protection against influenza A virus infection in BDP mice [89].

The roles that EARs play to support the innate immune response of the host against pathogens are not restricted to direct interactions with pathogens. Both hRNase 2 (EDN) and mEAR2 are chemotactic for dendritic cells (DCs) in vitro and in vivo [90, 91] while mEAR11 is chemotactic for macrophages [10] and neutrophils [92]. Injection of mEAR2 into the air pouches of mice triggered DC migration into the tissue. The capacity of those EARs to induce DC chemotaxis is related to p42/44 MAPK pathway activation. The chemotactic activity of mEAR2 is mostly attributed to its N‐terminal region as substitution of the 9 first amino acids of mEAR2 by the 10 first amino acids of human RNase 1, which possesses no chemotactic activity, impairs the capacity of mEAR2 to attract DCs. Given that EARs are mainly secreted in response to viral and parasitic infections, the chemotactic capacities of EDN and mEAR2 may contribute to increase antigen processing and presentation of DCs, therefore providing an optimal scenario to initiate specific immune responses [90]. EDN is also capable of activating DCs and enhancing T helper (Th)2‐biased immune responses through activating the Toll‐like receptor (TLR)2‐myeloid differentiation factor 88 pathway. Besides, mice DCs were also activated in the presence of EDN, indicating that the capacity to orchestrate immune responses by EARs may be conserved in mice [8]. mEAR11 also possesses chemotactic properties [10, 92]. Recombinant mEAR11 is capable of in vitro chemoattracting mouse‐isolated macrophages in a TLR2‐independent manner [10]. Besides, transgenic mice overexpressing mEAR11 presented increased neutrophil development and infiltration in tissues, while, in mEAR11 KO mice, this process was impaired [92]. The capacity of mEAR11 to chemoattract macrophages and neutrophils is apparently important during Th2 immune responses. Expression of mEAR11 by alveolar macrophages is induced by Th2‐related cytokines such as IL‐4 and IL‐13 [10, 42]. During a Th2 predominant response, chemokines responsible for the recruitment of immune cells during inflammatory responses, such as CXCL1 during neutrophil chemoattraction [93], are not produced by the alternatively associated macrophages (AAMs) [94, 95]. In this scenario, the participation of Th2‐related chemokines such as mEAR11 may be necessary to recruit immune cells to the infection area [92]. The exact neutrophil and macrophage receptors by which mEAR11 induces cell chemoattraction remain to be elucidated [10, 92].

On its side, EDN has been identified as a supplier, together with RNase T2, of uridine TLR8 ligands to initiate cytokines release and initiate immune responses. During infection, RNase 2 and RNase T2 work cooperatively in degrading pathogenic RNA in the endolysosomal compartment of monocytes and macrophages. While RNase 2 cleavage is produced after uridine and before purine, RNase T2 cleavage preference is after purine and before uridine, which enables the release of free uridines that, together with ssRNA fragments, can act as ligands to activate the TLR8‐Myd88 pathway [96, 97]. Whether mEARs can participate in an equivalent process mediated by TLR activation at endosomal level awaits investigation. However, significant differences between human and mice TLR8 specificities and functionalities [98] hinder a direct side‐by‐side comparison. On the other hand, there are currently humanised TLR7/TLR8 mouse models that can help to explore human eosinophil RNases action in vivo [99, 100].

### 1.3. RNase 4

RNase 4 is one of the RNase A superfamily members that has the highest interspecies similarities, with ~90% gene sequence identity. The high conservation of RNase 4 suggests that a strong evolutionary pressure exists to maintain its function, which may play a key role in host homeostasis [101]. Other unique traits of RNase 4 are that it constitutes the shortest polypeptide in the RNase A family and exerts strong cleavage specificity for the 3′ site of uridine, possessing 1000‐fold higher activity against poly(U) than poly(C) [102]. RNase 4 is widely expressed in the body, especially in the liver, with the exception of brain and placenta [103]. Both human and mouse RNase 4 genes share 5′ untranslated regions (5′‐UTRs) with angiogenin (Ang) and Ang‐1, respectively. Commonly, RNase A family genes simply consist of a small non‐coding exon 1 followed by an uninterrupted coding exon 2, with the promoters located upstream of exon 1. However, the RNase 4/Ang locus is constituted by four exons and three introns in both human and mouse, with its expression being controlled by two promoters that regulate gene expression with tissue specificity. The proximal promoter 2 (Pr2) contains a sequence for hepatocyte nuclear factor (HNF)‐1α binding, which enables the predominant expression of RNase 4 and Ang in the liver [104]. RNase 4 is also expressed in the gut by intestinal epithelial cells, mainly by Paneth and Goblet cells, and can be detected in human and mouse stool [105]. RNase 4 can also be detected in urine as it is expressed in the kidney collecting duct by intercalated cells (ICs) and bladder epithelium [106, 107]. Monocytes and macrophages could also contribute to increase RNase 4 levels in the urinary tract [108, 109].

RNase 4 has a key role in protecting the urinary tract against uropathogens. Both human and mouse RNase 4 demonstrate potent activity at low micromolar concentrations against uropathogenic *E. coli* (UPEC) [106, 107], which is the leading cause of bacterial urinary tract infections (UTIs) in humans. Urine RNase4 concentrations are reduced in women with recurrent UTI, indicating that altered expression of the *RNASE4* gene in the uroepithelium may be a risk factor. Besides, silencing of *RNASE4* or antibody neutralisation in vitro in primary human cultures of bladder urothelium and kidney medullary cells increases invasion and proliferation of UPEC [107]. Additional evidence for the importance of RNase 4 in host defence of the urinary tract has been generated in the context of diabetes mellitus (DM), a prevalent human condition associated with increased UTI susceptibility. Insulin and insulin receptor (IR) are important mediators of AMP expression in the urinary tract, including RNase A superfamily members [106, 110]. Recent findings reveal that kidney ICs require IR to trigger stress responses that activate the ATF4‐NFκB pathway and downstream induction of AMP expression [111]. Accordingly, selective deletion of β‐IR in renal IC of mice reduced the expression of RNase 4, along with other AMPs, thereby increasing UTI susceptibility. Furthermore, a type 2 DM mouse model exhibits increased UTI susceptibility, replicating the same phenotype observed in insulin‐resistant human patients. The relationship between DM and diminished RNase 4 expression was further confirmed in humans, as children with type 2 DM presented lower urinary median concentration of RNase 4 than healthy controls [106]. However, at this moment the lack of loss of function experiments with RNase 4 KO mice currently prevents us from accurately understanding the true importance of this protein in the protection of the urinary tract against infections. Besides, given that both urothelial cells and collecting duct ICs produce RNase 4 [106], the relative importance of each RNase 4 source in the urinary tracts remains to be elucidated.

Apart from its function in the urinary tract, RNase 4 also participates in host homeostasis in the gut. Specifically, studies in mice revealed that RNase 4 modulates the intestinal microbiome by selectively limiting *Parasutterella* growth while promoting the indoleamine‐2,3‐dioxygenase 1 (IDO1) pathway and reducing colitis risk. The IDO1 enzyme is secreted by intestinal epithelial and immune cells to convert tryptophan into downstream metabolites that contribute to gut homeostasis and prevent colitis. Control of *Parasutterella* growth by RNase 4 prevents the intestine from abnormal tryptophan metabolism. Recombinant mouse RNase 4 effectively kills *Parasutterella* in vitro by inducing damage to the bacterial cell wall at low micromolar concentrations. Interestingly, oral administration of the recombinant mouse RNase 4 in *RNASE4* ^*−/−*^ mice successfully ameliorates the symptoms of colitis and reduces *Parasutterella* population. The importance of RNase 4 in gut homeostasis is likely shared with humans as patients with inflammatory bowel disease (IBD) express diminished levels of RNase 4 in intestinal tissues and stool samples, which also correlates with increased prevalence of *Parasutterella* populations compared to healthy controls [105].

### 1.4. Angiogenins

Type 5 RNases, also named angiogenins (Ang) after their angiogenic properties [112], are considered the most structurally conserved RNase A subfamily with respect to the original family ancestor based on the fact that they are the closest mammalian ribonucleases to those found in non‐mammalian vertebrates. A particular characteristic of Ang subfamily, which is common to non‐mammalian vertebrate RNases, is the presence of six paired cysteine residues instead of four [1]. While humans only possess a single member of this subfamily, called Ang or RNase 5, the Ang subfamily experienced a rapid expansion in rodents. Accordingly, there are six functional Ang genes and three pseudogenes in mice, which share 72%–81% sequence identity [113]. Notably, although Ang3 was initially reported, sequence analysis indicates that its gene is present in the BALB/c mouse strain but absent from the C57BL/6J reference genome. The BALB/c Ang3 sequence aligns most closely, though not perfectly, with the Ang5 locus in the reference genome. mAng1 is the most closely related to hAng, sharing 77.5% sequence identity [20, 113]. Although the catalytic activity of hAng is low, an intact active site is necessary for its angiogenic function [114, 115]. Evolutionary studies of the Ang subfamily in primates revealed that its angiogenesis function is strongly conserved along evolution as the key residues related to this activity are positively selected [116, 117]. In mice, the selective pressure for maintaining angiogenic activity is not shared among all mAngs. While mAng1, mAng3 and mAng4 have angiogenic activities comparable to human Ang, mAng2 is not angiogenic [118–120]. To the best of our knowledge, there are no studies exploring activities for mAng5 and mAng6; however, it cannot be discarded that those proteins and mAng2 may have still unrevealed roles. Analysis of mutational dynamics of mAngs during evolutionary diversification suggests functional divergence among members of the subfamily, with functional constraints in amino acid positions changing after gene duplication [20]. Some of the additional forces of positive selection that may have generated functional diversity in mAngs are host defence roles. Most residues related to angiogenesis are located on the protein surface and could also mediate interactions with pathogens. The original function of the family ancestor remains unclear. Some authors suggest that the primal RNase A family ancestor likely possessed neither catalytic nor angiogenic capacities while being related to host defence [1]. In contrast, discovery of fish RNases with angiogenic properties supports an angiogenic origin, open to coexistence of shared angiogenic and antimicrobial properties [121, 122]. Similarly to what is observed in mEARs, fast sequence divergences and high rates of mutation are likely driven by selective pressures from microorganisms, leading to an increase in the antimicrobial arsenal available to counter emerging pathogens [123].

Given that hAng and mAng1 genes share promoters and 5′‐UTR regions with RNase 4, the expression levels and tissue distribution of both proteins are expected to be similar [104]. Both hAng and RNase4 are widely expressed in human tissues with almost identical patterns and distribution, with a clear enrichment in the liver but also present to a lesser extent in small intestine, heart, lung, skeletal muscle, pancreas, prostate, testis and ovary [45]. Besides, hAng can be detected in many fluids such as plasma [124], amniotic fluid [125], tumour microenvironment [126] and cerebrospinal fluid [127]. The abundant presence of hAng throughout the human body suggests that the role of this protein is not limited to angiogenesis [128]. The expression pattern of mAng1 is very similar to that of hAng as it is abundantly expressed in liver but also detectable in pancreas, lung and small intestine [129, 130]. mAng3 is present in lung and adult prostate while mAng4 is expressed in the small intestine by Paneth and goblet cells upon exposure to microbiota components or pathogens [129, 131, 132].

Multiple antimicrobial properties are attributed both to human and mice Angs. Although hAng possesses little antimicrobial activity against most common bacterial pathogens [129, 133], it was identified as an anti‐mycobacterial agent capable of killing *Mycobacterium tuberculosis* both extracellularly and in infected macrophages [134]. However, another study using recombinant hAng did not find any activity for this RNase in macrophages infected by nontuberculous mycobacteria, in contrast to other family members, as ECP and RNase 6 [135]. However, hAng is active against *Streptococcus pneumoniae* while not against *S. pyogenes* and also possesses a strong capacity to kill *Candida albicans*, indicating that it may have been evolutionarily specialised to limit the growth of specific pathogens [129, 133]. Similarly, mAng1 efficiently kills *C. albicans* and *S. pneumoniae* while mAng4 is much more active against *Enterococcus faecalis* and *Listeria monocytogenes* [129]. mAng4 also demonstrated high activity against *E. coli*, *S. thyphimurium*, *E. gallinarum* and *Bacteroides thetaiomicron* [131, 136]. This evidence supports the idea that host defence roles were at least one of the selective forces promoting Ang diversification in mice [1]. As abovementioned, hAng also possesses anti‐HIV activity and was identified as a soluble factor secreted by T cells to inhibit viral replication [137]. Besides, recombinant hAng reduces HIV burden during infection of phytohemagglutinin (PHA)‐stimulated T cell blasts [34]. To our knowledge, no study has reported antiviral activity for mAngs.

Studies using different mouse models of intestinal diseases revealed that mAngs are crucial in regulating microbiota composition and in maintaining gut homeostasis. The fact that mRNase 4 also plays a role in controlling gut bacterial population may be the functional reason explaining why mAng1 and mRNase 4 share the same promoter [104], as both RNases need to be secreted to the intestinal lumen. mAng1 limits the growth of α‐Proteobacteria strains, which can be colitogenic in gut dysbiosis conditions, while it favours the proliferation of *Lachnospiraceae* strains and prevents gut inflammation [130]. The fact that the levels of both hAng and mAng1 in stool samples are significantly lower in IBD patients and mice with colitis, respectively, suggests that hAng could also maintain microbial homeostasis in the human gut. Paneth cells are the main cell type secreting Ang in the intestine, and their deficiency is a common characteristic of IBD patients [130, 138]. Besides, hAng and mAng1 exert similar antimicrobial activities toward gut resident bacteria *in vitro*, with stronger activity against the Gram‐negative strains *Brevudimonas diminuta* and *Sphingomonas paucimobilis* than Gram‐positive strains *Anaerostirpes* sp. and *Blautia* sp. Ang specificity towards harmful Gram‐negative bacteria, while preserving Gram‐positive gut resident populations, may be explained by differences in bacterial membrane composition. While Gram‐negative bacteria have a highly anionic outer membrane enriched in lipopolysaccharide and a thin peptidoglycan wall, Gram‐positive bacteria lack an outer membrane and are surrounded by a much thicker peptidoglycan wall, which naturally confers more protection against AMPs [130]. mAng4 is also related to gut microbiome regulation. In response to pathogenic or commensal intestinal bacteria, crosstalk between intraepithelial lymphocytes (iELs) and enterocytes is initiated, triggering the secretion of mAng4 by Paneth cells. The detection of *Salmonella* and components of the microbiota by enterocytes triggers the secretion of IL‐23, which activates iELs to induce the secretion of IL‐22 that finally activates mAng4 secretion by Paneth cells. Once secreted, mAng4 can inhibit pathogen invasion and maintain the intestinal microbial homeostasis [131]. Besides, mAng4 promotes the abundance of beneficial bacterial populations, such as *Lactobacillus* or *Ackermansia*, while reducing pathogenic bacterial populations such as *Alistipes* and *Enterohabdus*. Therefore, mAng4 apparently selects bacterial populations that have anti‐inflammatory capacities [136]. Lastly, mAng4 is likely to participate in the expulsion of the gastrointestinal nematode *Trichuris muris*, as deduced from the protein expression profile pattern during infection [132].

### 1.5. RNase 6

RNase 6 lineage is stable in both human and mouse compared to closely related RNases such as EARs [139, 140]. The rodent RNase 6 ancestor was subjected to strong evolutionary forces before separation of *Rattus* and *Mus* genera, while in the more recent evolution rodent *RNASE6* genes are more conserved [140]. Despite the presence of *RNASE6* gene expansion in some rodent species, with duplication events leading to additional and divergent copies, mouse only possesses a single *RNASE6* gene as in humans [141]. Mice and human RNase 6 sequences share a 68% identity. The mRNase 6 possesses 17 times more catalytic activity than the human counterpart, when assayed using recombinant proteins and tRNA as a substrate [140]. The human *RNASE6* transcript is broadly present in tissues, with detection in all tested organs and predominance in lung, placenta, heart and kidney [142]. The mouse *RNASE6* transcript was detected in heart, spleen, thyroids and salivary glands [140]. Both human and mouse RNase 6 are expressed by monocytes, macrophages and B lymphocytes with high transcript levels in spleen and thymus [142–144]. The fact that RNase 6 protein is not detected at baseline in human CD14 (+) monocytes and murine bone‐marrow derived macrophages (BMDMs) may be indicative of its post‐transcriptional regulation. RNase 6 can also be detected in intracellular granules of mouse myeloid cells, indicating that the protein may be stored until its secretion is induced by bacteria, a mechanism shared with EARs [143].

High antibacterial properties for RNase 6 have been reported. In vitro incubation of hRNase 6 or mRNase 6 with Gram‐negative and positive bacteria at micromolar concentrations leads to a dose‐dependent rapid killing of microbes, including uropathogenic strains of *E. coli*, *E. faecalis* and *S. saprophyticus* [143, 145]. RNase 6 is an important host defence protein in the kidney and lower urinary tract. In response to UTI, RNase 6 is secreted by infiltrated monocytes and macrophages to control the infection [143, 144, 146]. In both human and mouse, RNase 6 levels are increased in response to pyelonephritis, and the antimicrobial protein can only be detected in urine following UPEC infection. Bacterial exposure is necessary to induce RNase 6 production by monocytes and macrophages [143]. Apart from being secreted, RNase 6 can perform its antimicrobial action within the lysosomal compartment of macrophages and enhance macrophage clearance of phagocytosed bacteria [143, 144, 146]. RNase 6 KO mice are more susceptible to UPEC infection while RNase 6 KO macrophages exhibit impaired intracellular UPEC killing [144]. Transgenic expression of hRNase 6 in mice leads to increased UPEC clearance during experimental cystitis [146]. A common non‐synonymous polymorphism in human *RNASE6* results in an arginine to glutamine transversion and altered antimicrobial activity toward UPEC, likely as a consequence of decreased surface charge and bacterial agglutination properties [147]. It remains to be determined whether *RNASE6* genetic variation influences human UTI risk.

Very recent results pointed out a novel role for RNase 6 in the cleavage of pathogen ssRNA and generation of specific uridine‐end products that activate TLR7/8 in endolysosomes [148, 149].

### 1.6. RNase 7

*RNASE7* exists exclusively in primates and no orthologous gene is present in mice [143]. This protein is secreted by many epithelial tissues and is present in skin, heart, liver, kidney, bladder, pharynx and tonsil [150–153]. In addition to constitutive RNase 7 expression in these tissues, RNase 7 can also be induced by a variety of stimuli, such as presence of commensal and pathogenic microorganisms [153–158], EGFR signalling [158, 159], pro‐inflammatory cytokines [160, 161], insulin‐mediated activation of the PI3K/AKT pathway [110], UV stimulation [162] and cigarette smoke exposition [158]. In the skin, RNase 7 is secreted by differentiated keratinocytes to control microbial growth and prevent bacterial, fungal and viral infections [133, 163]. RNase 7 demonstrates potent activity in vitro at low micromolar concentrations against skin pathogens such as *S. aureus*, *P. aeruginosa*, *C. albicans* and *Trichophyton rubrum* among others [133, 156, 160, 164]. Apart from exerting direct antimicrobial action, RNase 7 is capable of binding to self‐DNA released from skin lesions to induce the expression of IFN‐related genes by plasmacytoid DCs and keratinocytes and initiate anti‐herpes simplex virus (HSV)‐1 responses [9, 163]. Notably, RNase 7 ability to inhibit HSV‐1 does not rely solely on IFN‐mediated responses; it also directly restricts viral replication in keratinocytes by reducing early gene expression of the virus and promoting degradation of incoming viral particles [165]. Furthermore, RNase 7 is upregulated in the genital mucosa of HIV‐exposed seronegative individuals, suggesting a protective role in preventing HIV infection [166].

RNase 7 activity is modulated by host factors that inhibit its enzymatic and antimicrobial activities. In atopic dermatitis, elevated RNH1 levels and accumulated extracellular self‐DNA from damaged cells neutralise RNase 7, impairing its antibacterial and antiviral activities and contributing to susceptibility to *S. aureus* and HSV‐1 [167, 168]. In contrast, reduced RNH1 expression during UTI may enhance RNase 7‐mediated defence [169].

Despite the absence of a murine *RNASE7* orthologue, humanised RNase 7 transgenic mice enabled the demonstration that this antimicrobial protein can protect the urinary tract from bacterial infection. Similarly to RNase 4, RNase 7 is secreted by bladder urothelium and the kidney’s ICs to prevent colonisation and infection caused by bacterial pathogens [106, 153]. RNase 7 also possesses strong activity in vitro against uropathogenic strains of *E. coli*, *E. faecalis and S. saprophyticus* species [143]. Like RNase 4, RNase 7 expression in the urinary tract is also under control of insulin signalling, and diabetic patients express diminished RNase 7 protein—further indicating that both proteins have very similar roles and expression patterns in the urinary tract [106, 110]. Besides, RNase 7 expression, together with RNase 4 and RNase 6, may be regulated through chromatin remodelling, since histone deacetylase (HDAC) inhibitors lead to increased expression by urothelial cells [170] and type II pneumocytes [171].

### 1.7. RNase 8

RNase 8 was the last canonical member of human RNase A superfamily to be characterised. *RNASE8* is most closely related to *RNASE7* and similarly exists exclusively in primates, indicating that a recent gene duplication event is the most plausible origin [172]. The physiological role of RNase 8 remains unclear. The protein shows specific structural traits with a peculiar cysteine distribution [173] that is unique within the RNase A superfamily along with an unusual pattern of diversification, with the presence of potential “functional pseudogenes” [172]. The protein expression was initially reported in placenta and later identified in lung, spleen and testis [172, 174] with a broad‐spectrum antimicrobial activity [33, 175]. However, its potential mechanism of action is unknown [176], and genetic studies suggest a significant divergence from the other seven canonical RNases [172].

## 2. Discussion

**Figure 4:**
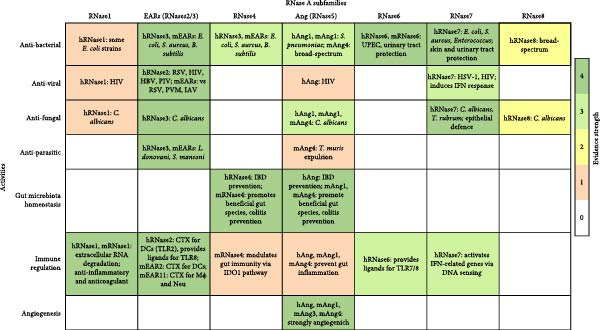
Functional activities of human and mouse RNase A superfamily members. Colour intensity reflects the strength of experimental evidence supporting each RNase activity. Evidence scores are categorised as: 0 = no evidence, 1 = weak evidence, 2 = moderate evidence, 3 = strong evidence, 4 = very strong evidence. Ang, angiogenin; CTX, chemotaxis; DC dendritic cells; EAR, eosinophil associated RNases; IBD, intestinal bowel disease; IDO1, indoleamine 2,3′ dioxygenase 1; Mɸ, macrophages; Neu, neutrophils.

Mouse models are powerful tools to study the roles of RNases during infection processes in vivo. Comparison of the chromosomal locations of human and mouse RNase genes highlights a conserved distribution pattern, with a striking expansion of members and presence of pseudogenes in mice (Figure 3). RNases 1, 4 and 6 are well conserved not only in terms of sequence identity, being 69.35%, 82.20% and 68.25%, respectively, but also with regard to their tissue distribution and function [27, 105, 143]. However, the expansion of mEAR and mAng genes observed in the mouse probably generated functional divergences between these families and their human orthologues, with reduced identity to 50% for some mouse RNases with respect to their human counterparts. For hEARs, a clear specialisation of EDN and ECP to antiviral and antibacterial functions, respectively, is exhibited [35]. In contrast, mEARs constitute a set of proteins with unknown level of specialisation of each RNase for particular pathogens or their activation expression profile [1, 21]. Besides, the physiological differences that exist between human and mouse eosinophils may also lead to functional differences in mEARs [59, 177]. Similarly, while mAng1 is apparently the closest to hAng, it is unclear if other mAngs have different roles such as antimicrobial specialisation [113, 129]. The reported biological properties of human and mice RNases, according to their subfamily type, have been summarised in Figure 4.

In addition, to further explore the contributions of RNases to host defence, we have summarised the current information regarding their expression patterns. Table 1 summarises the expression changes based on transcriptomic data collected from the *EMBL-EBI Expression Atlas*.

**Table 1:** Expression profile of human and mouse RNases in infection.

RNase type	Infection conditions and expression changes
1	**hRNase1** • ↑During meningococcal sepsis at 24 h vs normal at 0 h in lymphocytes • ↓During *Mycobacterium tuberculosis* Inf. ⚬ Inf. vs Ctrl. in adult ⚬ Inf. vs Ctrl. in infant ⚬ Purified protein derivative (PPD) vs Ctrl. during tuberculosis active infection ⚬ Inf. vs Ctrl. in macrophage and dendritic cells at 18 and 48 h • ↑During hepatitis B virus (HBV) associated acute liver failure vs Ctrl. • ↑During leishmaniosis vs normal **mRNase1** • ↓During severe acute respiratory syndrome coronavirus (SARS‐CoV) MA15 ⚬ Inf. at day 2 vs Ctrl. in NOD/ShiLtJ ⚬ Inf. at day 4 vs Ctrl. in PWK/PhJ • ↑During acute oropharyngeal candidiasis vs Ctrl. in wild type mice

2/3	**hRNase2 (EDN)** • ↑During lymphocyte meningococcal sepsis at 8 and 24 h vs Ctrl. at 0 h • ↑During meningococcal sepsis at 24 h vs Ctrl. at 0 h in blood • ↓During *M. tuberculosis* Inf. ⚬ Inf. vs Ctrl. in infant and adult ⚬ Inf. vs Ctrl. in macrophage at 48 h • ↑During *M. tuberculosis* Inf. ⚬ Inf. vs Ctrl. ⚬ Inf. and treatment for 3 and 6 months vs Ctrl. • ↑During septic shock (group B streptococcus) vs Ctrl. • ↓During *Staphylococcus aureus* Inf., *s*trains 10,254 and Col at 6 h vs Ctrl. at 0 h • ↑During COVID‐19 Inf. vs Ctrl. in lung • ↑Hepatitis C (HCV) patients, peginterferon alfa‐2a treatment at 16 h vs Ctrl at 0 h in biopsies • ↑During HBV associated liver failure vs Ctrl. • ↑During Respiratory syncytial virus (RSV) Inf. vs Ctrl. • ↑During unidentified influenza virus Inf. vs Ctrl. • ↑During Leishmaniasis vs Ctrl. **hRNase3 (ECP)** • ↑During bacteraemia (*Staphylococcus aureus* and *Escherichia coli*) vs Ctrl. • ↑During *Listeria monocytogenes* Inf. vs Ctrl. • ↑During Lyme disease acute phase of Inf. prior to initiation of antibiotic treatment vs Ctrl. • ↑During mechanical ventilation and ventilator‐associated pneumonia vs Ctrl. • ↑During Meningococcal sepsis ⚬ Inf. at 8 and 24 h vs Ctrl. at 0 h in monocytes ⚬ Inf. at 24 h vs Ctrl. at 0 h in blood and lymphocytes • ↑During septic shock (group B streptococcus) vs Ctrl. • ↓During *S. aureus* Inf., strain 252 and 1025 at 6 h vs Ctrl. at 0 h • ↑During tuberculosis treatment with 2HRZE/4HR at 4 weeks vs Ctrl. at 0 week • ↑During RSV Inf. vs Ctrl. • ↑During unidentified influenza virus Inf. vs Ctrl. • ↑During Leishmaniosis vs Ctrl. **mEAR1** • ↓During *M. tuberculosis* Inf. ⚬ Inf. vs Ctrl. in TNF‐α KO ⚬ TNF‐α 250 microgram vs Ctrl. in WT ⚬ Inf. at day 30 vs Ctrl. at day 0 ⚬ Inf. at days 30 and 70 vs Ctrl. at day 0 in DBA/2J congenic ⚬ Inf. at day 70 vs Ctrl. at day 0 in congenic D2.B6‐Chr19/D2.B6‐Chr7 and C57BL/6J • ↑During *S. aureus* Sanger 476 Inf. ⚬ Inf. at 2 h vs Ctrl. at 0 h in C57BL/6J ⚬ Inf. at 2, 6 and 12 h vs Ctrl. at 0 h in A/J • ↓During Influenza A virus (IAV) Inf. ⚬ Inf. vs Ctrl. ⚬ A/Puerto Rico/8/1934(H1N1) Inf. at day 4 vs Ctrl. in WSB/EiJ, PWK/PhJ and NOD/ShiLtJ • ↓During SARS‐CoV MA15 Inf. ⚬ Inf. at day 2 vs Ctrl. in WSB/EiJ ⚬ Inf. at days 2 and 4 vs Ctrl. in PWK/PhJ ⚬ Inf. at days 2 and 4 vs Ctrl. in NOD/ShiLtJ ⚬ Inf. at day 4 vs Ctrl. in WSB/EiJ and C57BL/6J • ↓During Sendai virus Inf. ⚬ Inf. vs UV‐inactivated virus at day 21 on Affymetrix MOE430A Array ⚬ Inf. vs UV‐inactivated virus at day 49 on Affymetrix Mouse430_2 Array • ↓During *Leishmania donovani* Inf. at 48 h vs Ctrl. at 48 h in 14M1.4 cells **mEAR2** • ↑During *Salmonella enterica* serovar Typhimurium Inf. vs Ctrl. in bone marrow macrophage from 129P2 • ↓During *Yersinia pestis* Inf. vs Ctrl. in neutrophil • ↓During IAV Inf. ⚬ Inf. vs Ctrl. ⚬ A/Puerto Rico/8/1934(H1N1) Inf. at day 4 vs Ctrl. in 129S1/SvlmJ and wSB/EiJ • ↓During SARS‐CoV MA15 Inf. at days 2 and 4 vs Ctrl. in PWK/PhJ, WSB/EiJ and NOD/ShiLtJ • ↓During *Debaryomyces hansenii* Inf. at 12 h vs Ctrl. at 0 h • ↑During *Nippostrongylus brasiliensis* Inf. vs Ctrl. • ↑During *Schistosoma*‐induced pulmonary hypertension vs Ctrl. **mEAR5** • ↑During *S. aureus* cutaneous Inf. vs Ctrl. in IL‐1R KO • ↓During acute oropharyngeal candidiasis, anti‐IL‐17A and anti‐IL‐17F treatment vs Ctrl. in WT • ↑During acute oropharyngeal candidiasis vs Ctrl. in WT **mEAR6** • ↑During IAV Inf. ⚬ Inf. vs none ⚬ Strain A/Puerto Rico/8/1934(H1N1) Inf. at day 4 vs Ctrl. in C57BL/6J • ↓During SARS‐CoV MA15 Inf. ⚬ Inf. at day 2 vs Ctrl. in CXCR3 KO ⚬ Inf. at days 2 and 4 vs Ctrl. in 129S1/SvImJ ⚬ Inf. at day 5 vs Ctrl. in STAT1 −/− ⚬ Inf. at day 5 in STAT1 −/− vs Ctrl. in WT ⚬ Inf. at day 7 vs Ctrl. in WT, TIMP1 KO, PAI1 KO, nsp16 −/− and Tnfrsf1a/1b −/− • ↑During SARS‐CoV MA15 Inf. ⚬ Inf. with E protein mutant Δ5 vs Ctrl. ⚬ Inf. with mutant lacking full‐length E protein vs Ctrl. ⚬ Inf. at days 2 and 4 vs Ctrl. in C57BL/6J • ↓During *Plasmodium chabaudi* Inf. vs Ctrl. in adjuvant only • ↑During *P. chabaudi* Inf. vs Ctrl. in vaccination **mEAR10** • ↓During IAV A/Puerto Rico/8/1934(H1N1) Inf. at day 4 vs Ctrl. in WSB/EiJ and PWK/PhJ • ↓During SARS‐CoV MA15 Inf. at days 2 and 4 vs Ctrl. in PWK/PhJ, WSB/EiJ and NOD/ShiLtJ **mEAR11** • ↓During *M. tuberculosis* Inf. at day 30 vs Ctrl. at day 0 in congenic D2.B6‐Chr19 • ↑During *S. aureus* Inf. ⚬ Inf. with MW2 at 2 h vs Ctrl. at 0 h in A/J ⚬ Inf. with USA300 at 2 h vs Ctrl. at 0 h in CD1 ⚬ Inf. with Sanger476 at 2 h vs Ctrl. at 0 h in BALB cByJ ⚬ Inf. with Sanger476 at 4, 6 and 12 h vs Ctrl. at 0 h in A/J and C57BL/6J • ↓During IAV Inf. vs Ctrl. in IgG1 • ↑During IAV A/Puerto Rico/8/1934(H1N1) Inf. at day 2 vs Ctrl. in A/J • ↓During SARS‐CoV MA15 Inf. ⚬ Inf. at day 2 vs Ctrl. in CXCR3 KO ⚬ Inf. at day 4 vs Ctrl. in WT, TLR3 KO and PWK/PhJ ⚬ Inf. at day 5 vs Ctrl. in STAT1 −/− ⚬ Inf. at day 7 vs Ctrl. in WT, Tnfrsf1a/1b −/−, nsp16 −/−, PAI1 KO and TIMP1 KO • ↑During SARS‐CoV MA15 Inf. ⚬ Inf. at day 4 vs Ctrl. in CAST/EiJ ⚬ Inf. at day 9, STAT1 −/− and IFNAR1 −/− vs WT • ↑During sendai virus Inf. vs UV‐inactivated at day 49 on Affymetrix Mouse430_2 Array • ↑During *P. chabaudi* Inf. vs Ctrl. in vaccination and adjuvant only • ↑During *Trypanosoma brucei* strain TREU927 Inf. vs Ctrl. • ↓During *N. brasiliensis* Inf. at day 8, BALB c SCID vs WT • ↑During Schistosoma‐ induced pulmonary hypertension vs Ctrl. **mEAR14** • ↑During IAV (A/Puerto Rico/8/1934(H1N1) Inf. at day 2 vs Ctrl. in NOD/ShiLtJ • ↑During SARS‐CoV MA15 day 2 Inf. vs Ctrl. in WSB/EiJ

4	**hRNase4** • ↑During *Salmonella enterica* subsp. enterica serovar Typhimurium Inf. vs Ctrl. • ↓During visceral leishmaniasis vs Ctrl. **mRNase4** • ↓During *S. enterica* serovar Typhimurium Inf. vs Ctrl. in bone marrow macrophage from 129P2 • ↑During *S. aureus* Sanger476 Inf. at 2 h vs Ctrl. at 0 h in C57BL/6J • ↓During IAV Inf. ⚬ Inf. with A/Scotland/20/1974(H3N2) vs Ctrl. ⚬ Inf. with severe influenza with IL‐22 treatment vs Ctrl. ⚬ Inf. with A/Puerto Rico/8/1934(H1N1) at day 4 vs Ctrl. in 129S1/SvImJ and IgG1 • ↓During Lymphocytic choriomeningitis virus (LCMV) Inf. vs none in WT and TRAIL KO • ↓During SARS‐CoV MA15 Inf. ⚬ Inf. with E protein mutant Δ3 vs Ctrl. ⚬ Inf. with E protein mutant Δ5 vs Ctrl. ⚬ Inf. at day 1 vs Ctrl. ⚬ Inf. at day 2 vs Ctrl. in WT, PWK/PhJ, nsp16 −/−, CAST/EiJ, C57BL/6J, WSB/EiJ and CXCR3 KO ⚬ Inf. with dORF6 at day 2 and 4 vs Ctrl. ⚬ Inf. at day 4 vs Ctrl. in WT, PWK/PhJ, Tnfrsf1a/1b −/−, nsp16 −/−, TLR3 KO, C57BL/6J, PAI1 KO and TIMP1 KO ⚬ Inf. at day 5 vs Ctrl. in STAT1 −/− ⚬ Inf. at day 5, STAT1 −/− vs WT ⚬ Inf. at day 7 vs Ctrl. in WT, PAI1 KO, TIMP1 KO and PLAT KO ⚬ Inf. with nsp16 −/− at day 7 vs Ctrl. • ↑During Theiler’s encephalomyelitis virus (TMEV) Inf. at day 196 vs Ctrl. • ↑During West Nile virus (WNV) Inf., late‐stage encephalitis vs early‐stage encephalitis • ↓During acute oropharyngeal candidiasis vs Ctrl. in WT • ↓During *D. hansenii* Inf. at 12 h vs Ctrl. at 0 h • ↓During *P. chabaudi* Inf. vs Ctrl. in only adjuvant and vaccination • ↓During *L. donovani* Inf. at 48 h vs Ctrl. in 14M1.4 • ↓During *Trichuris muris* worm separated and left Inf. vs Ctrl. in caecum • ↑During Right middle lobe (RML) syndrome ⚬ Inf. at 7 weeks vs Ctrl. in B4053 ⚬ Inf. at 22 weeks vs Ctrl. in FVBNCr ⚬ Inf. at 56 weeks vs Ctrl. in FVBPrnp01 • ↑During murine prion 301V Inf. at 16 weeks vs Ctrl. in B6I1

5	**hRNase5 (ANG)** • ↑During meningococcal sepsis Inf. at 24 h vs Ctrl. at 0 h in blood • ↓During *M. tuberculosis* Inf. ⚬ Inf. vs Ctrl. in adult ⚬ Purified protein derivative (PPD) vs Ctrl. • ↑During *M. tuberculosis* Inf. vs Ctrl. at 48 h in macrophage • ↓During *S. aureus* strain SK2 Inf. vs Ctrl. • ↓During HBV‐associated liver failure vs Ctrl. • ↓During HCV Inf., treatment with acetaminophen vs none in HepaRG **mANG1** • ↓During *S. enterica* serovar Typhimurium Inf. vs Ctrl. in bone marrow macrophage from 129P2 • ↓During IAV A/Scotland/20/1974(H3N2) Inf. vs Ctrl. • ↓During SARS‐CoV MA15 Inf. ⚬ Inf. at day 2 vs Ctrl. in WT, PWK/PhJ, CAST/EiJ, C57BL/6J, TLR3 KO and nsp16 −/− ⚬ Inf. with dORF6 at days 2 and 4 vs Ctrl. in WT ⚬ Inf. at day 4 vs Ctrl. in WT, PWK/PhJ, PAI1 KO and TIMP1 KO ⚬ Inf. at day 7 vs Ctrl. WT and PAI1 KO • ↑During WNV late‐stage encephalitis vs Ctrl. • ↓During *D. hansenii* at 6 and 12 h Inf. vs Ctrl. at 0 h • ↓During *P. chabaudi* Inf. vs Ctrl. in only adjuvant and vaccination • ↓During *T. muris* worm separated and worm left Inf. vs Ctrl. in caecum • ↑During murine prion 301V Inf. at 16‐week vs Ctrl. in B6I1 • ↑ During Right middle lobe (RML) syndrome Inf. at 16‐week vs Ctrl. in BL6 **mANG2** • ↓During IAV Inf. ⚬ Inf. with A/Scotland/20/1974(H3N2) vs Ctrl. ⚬ Inf. with A/Puerto Rico/8/1934(H1N1) at day 4 vs Ctrl. in NOD/ShiLtJ and PWK/PhJ • ↓During SARS‐CoV MA15 Inf. ⚬ Inf. at day 1 vs Ctrl. ⚬ Inf. at day 2 vs Ctrl. in WT, WK/PhJ, nsp16 −/−, TLR3 KO and CXCR3 KO ⚬ Inf. with dORF6 at days 2 and 4 vs Ctrl. ⚬ Inf. at day 4 vs Ctrl. in WT, PWK/PhJ, WSB/EiJ, NOD/ShiLtJ and Tnfrsf1a/1b −/− ⚬ Inf. at day 7 vs Ctrl. in WT and PAI1 KO • ↓During *L. donovani* 48 h Inf. vs Ctrl. at 48 h in 14M1.4 • ↓During *P. chabaudi* Inf. vs Ctrl. in vaccination and adjuvant only **mANG4** • ↓During IAV Inf. ⚬ Inf. with A/Scotland/20/1974(H3N2) vs Ctrl. ⚬ Inf. with A/Puerto Rico/8/1934(H1N1) at day 2 vs Ctrl. in NOD/ShiLtJ ⚬ Inf. with A/Puerto Rico/8/1934(H1N1) at day 4 vs Ctrl. in NOD/ShiLtJ, WSB/EiJ and PWK/PhJ • ↓During SARS‐CoV MA15 Inf. ⚬ Inf. at day 2 vs Ctrl. in WT, PWK/PhJ, WSB/EiJ, NOD/ShiLtJ, TLR3 KO, nsp16 −/− and CXCR3 KO ⚬ Inf. with dORF6 at days 2 and 4 vs Ctrl. ⚬ Inf. at day 4 vs Ctrl. in WT, PWK/PhJ, WSB/EiJ, NOD/ShiLtJ, Tnfrsf1a/1b −/− and PAI1 KO ⚬ Inf. at day 7 vs Ctrl. in WT and PAI1 KO • ↓During *L. donovani* Inf. at 48 h vs Ctrl. at 48 h in 14M1.4 • ↓During *P. chabaudi* Inf. vs Ctrl. in vaccination and adjuvant only • ↑During *N. brasiliensis* Inf. vs Ctrl. • ↑During *T. muris* worm separated and worm left Inf. vs Ctrl. in caecum **mANG5** • ↓During IAV A/Puerto Rico/8/1934(H1N1) Inf. at day 4 vs Ctrl. in WSB/EiJ and PWK/PhJ • ↓During SARS‐CoV MA15 Inf. at days 2 and 4 vs Ctrl. in PWK/PhJ, WSB/EiJ and NOD/ShiLtJ

6	**hRNase6** • ↓During *Francisella tularensis* Novicida and Schu S4 Inf. vs Ctrl. • ↓During Meningococcal sepsis at 0 h vs Ctrl. at 0 h in monocyte • ↑During meningococcal sepsis at 8 and 24 h vs Ctrl. at 0 h in lymphocyte • ↓During *M. tuberculosis* Inf. ⚬ H37Rv whole cell lysate vs Ctrl. in pulmonary and meningeal tuberculosis ⚬ Inf. vs Ctrl. in infant and adult ⚬ Purified protein derivative (PPD) vs Ctrl. ⚬ Inf. at 18 and 48 h vs Ctrl. in DC and macrophage. • ↑During *M. tuberculosis* Inf. ⚬ Inf. vs Ctrl. ⚬ Treatment for 3 months vs Ctrl. • ↓During septic shock (group B streptococcus) vs Ctrl. • ↓During *S. aureus* Inf. ⚬ Inf. with 11,490 at 3 h vs Ctrl. at 0 ⚬ Inf. with 252, 10,254, 9897 and Col at 3 and 6 h vs Ctrl. at 0 h • ↓During HBV Inf., immune tolerance phase vs immune clearance phase • ↑During HBV Inf., associated acute liver failure vs Ctrl. • ↓During HCV JFH1 Genotype 2A Inf., IL28B 10 U/mL vs Ctrl. in primary human hepatocytes • ↑HCV patients, peginterferon alfa‐2a treatment (180 μM) at 144 h vs none at 0 h in biopsies • ↓During human Cytomegalovirus TB40E TB and Towne/E Inf. vs Ctrl. in monocytes • ↓During human gamma herpes virus 4 Inf. at days 1, 2 and 3 vs Ctrl. at day 0. • ↓During Influenza A virus (IAV) H1N1 Inf. ⚬ Stimulated with single‐stranded oligonucleotides vs Ctrl. in monocyte‐derived DCs. ⚬ Stimulated with Poly I:C vs Ctrl. in monocyte derived DCs. • ↑During IAV H1N1 Inf., single‐stranded oligonucleotides + Poly I:C vs Ctrl. in monocyte derived DCs • ↓During Newcastle disease virus Inf. at 14, 16 and 18 h vs Ctrl. at 0 h • ↑During Pick disease Inf. vs Ctrl. **mRNase6** • ↓During *Bifidobacterium bifidum* Z9 Inf. vs Ctrl. • ↓During *Helicobacter hepaticus* Inf. ⚬ Inf. vs Ctrl. in WT ⚬ Inf. vs Ctrl. in Alpk1 KO • ↓During *Lactobacillus acidophilus* Inf. ⚬ Inf. with NCFM vs Ctrl. ⚬ Inf. with NCFM and *Bifidobacterium bifidum* Z9 vs Ctrl. • ↑During *M. tuberculosis* Inf. ⚬ Inf. with H37Rv vs Ctrl. in WT ⚬ Inf. at day 30 vs Ctrl. ⚬ Inf. at days 30 and 70 vs Ctrl. at day 0 in D2.B6‐Chr19 congenic, D2.B6‐Chr7 congenic, DBA/2J congenic and C57BL/6J ⚬ Inf. at day 70 vs Ctrl. • ↓During *S. enterica* serovar Typhimurium Inf. vs Ctrl. in derived macrophage from E14 Traf2 mutant cell line • ↑During *S. enterica* serovar Typhimurium Inf. vs Ctrl. in derived macrophage from E14 cell line and bone marrow macrophage from 129P2 mouse • ↓During *S. aureus* Sanger476 Inf. ⚬ Inf. at 2 h vs Ctrl. at 0 h in A/J and C57BL/6J ⚬ Inf. at 4 h vs Ctrl. at 0 h in C57BL/6J • ↑During Chikungunya virus Inf., late‐stage encephalitis vs Ctrl. • ↑During IAV Inf. ⚬ Inf. vs Ctrl. in IL‐22 ⚬ Inf. vs Ctrl. in IgG1 ⚬ Inf. with A/Puerto Rico/8/1934(H1N1) at day 2 vs Ctrl. in NOD/ShiLtJ and 129S1/SvImJ ⚬ Inf. at days 2, 3 and 4 vs Inf. at day 1 in DBA/2J ⚬ Inf. with A/Puerto Rico/8/1934(H1N1) at day 4 vs Ctrl. in 129S1/SvImJ, PWK/PhJ, NOD/ShiLtJ, NZO/HILtJ, WSB/EiJ and A/J • ↓During LCMV Inf. vs Ctrl. in TRAIL homozygous KO • ↓During SARS‐CoV MA15 Inf. at days 5 and 9 in STAT1 −/− vs WT • ↑During SARS‐CoV MA15 Inf. ⚬ Inf. with E protein mutant Δ5 vs Ctrl. ⚬ Inf. with virus lacking full‐length E protein vs Ctrl. ⚬ Inf. at day 2 vs Ctrl. in 129S1/SvImJ, WSB/EiJ and PWK/PhJ ⚬ Inf. at day 4 vs Ctrl. in PWK/PhJ, WSB/EiJ A/J and 129S1/SvImJ ⚬ Inf. at day 5 vs Ctrl. in WT • ↑During TMEV infection at days 42 and 98 vs Ctrl. • ↓During *D. hansenii* Inf. at 12 h vs Ctrl. at 0 h • ↑During *P. berghei* Inf. ⚬ Inf. with ANKA at day 6 vs Ctrl. at day 0 ⚬ Inf. at day 7, artesunate and chloroquine vs none • ↑During *T. muris* worm separated and worm left Inf. vs Ctrl. in caecum

7	**hRNase** **7** • ↑During Neisserial ligand MafA exposition vs Ctrl. • ↓During HCV JFH1 Genotype 2A Inf., treatment with IL28B vs Ctrl. in primary human hepatocytes • ↑During human cytomegalovirus (Towne strain) Inf. vs Ctrl. • ↑During live scabies mite Inf. vs Ctrl.

*Note:* The data are summarised from Expression Atlas [178] (https://www.ebi.ac.uk/gxa/home). Only infection related conditions are included, using the search terms “infectious disease”, “bacterial disease”, “viral disease”, “fungal infectious disease” and “parasitic infection” for all mouse RNases genes present in the table. Alternatively, additional searches were performed individually from every infection‐related experiment entry using terms for all human or mouse RNases. Ang6 was excluded because it does not exhibit significant expression changes, whereas Ang3 was omitted since it is absent from the C57BL/6J reference genome and therefore not represented in the database. Up and downregulation is indicated by arrows. Human and mouse RNases are grouped according to respective subfamily types. Infection models are ordered according to pathogen type. Inf. refers to infection conditions while Ctrl. refers to the respective control.

These data reveal that RNases genes can be both up and downregulated during infection. Given that RNase A superfamily members exert antimicrobial activity, we hypothesise that downregulation of RNase expression may be triggered by some pathogens as part of an immunosuppressive strategy to counter the host immune response. Downregulation of AMP expression during the course of infection is a strategy followed by some pathogens to successfully invade the host [179]. For example, it is well known that *M. tuberculosis* is capable of promoting a M1 to M2 shift in infected macrophages to induce the production of anti‐inflammatory effectors and create a permissive environment for growth and persistence. Besides, the high heterogeneity found in lung macrophages may lead to different inflammatory states in individual cells, therefore providing inconclusive results unless using single‐cell transcriptomics techniques [180]. Viral pathogens such as coronavirus and influenza virus also possess mechanisms to impair the host’s immune response, mainly based on blunting interferon signalling [181, 182]. The infection timing is another factor that can lead to different inflammatory profiles. For example, during COVID‐19 infection an early immunosuppressive stage occurs while the late stage of the infection involves an activated immune response with subsequent chemokine storm and lung damage [183]. In cell line studies, THP‐1 macrophages exposed to recombinant human ECP revealed an early pro‐inflammatory response independent of catalytic activity followed by the activation of specific antiviral pathways in a catalytic‐dependent manner [43]. Comparison of expression patterns in both human and mice infections (Table 1) reveals an overall similar pattern, with more disparity for mice proteins. When analysing human RNase expression profiles according to infection type, we observe a predominant increase associated with sepsis (hRNases 1, 2, 3, 5), bacterial infection (hRNases 3, 6) and viral infection (hRNase2). Another clear tendency drawn from the data is the downregulation of RNases at the onset of tuberculosis infection with a significant induction after long‐term (>30 days) exposure. However, the current available data are very incomplete, and no direct conclusions can be extracted in relation to infection type, timing and conditions, due to lack of experimental data registered in equivalent conditions for mice and men. Besides, although most human and murine pathogens share similar features, significant differences arise for some species and conditions, such as coronavirus disease [184], which demand a deeper analysis. In addition, there are significant differences at the TLR sensing system that aroused from distinct specific host–pathogen co‐evolution processes in human and rodents [185], which limits a straightforward comparison. Last, together with transcriptomics, we need to consider the changes in secreted RNases at the protein level, along with any changes in post‐translational modifications following pathogen exposure. This is particularly important for cases such as EAR RNases, where eosinophil degranulation might be the key triggering step following pathogen exposure.

**Figure 5:**
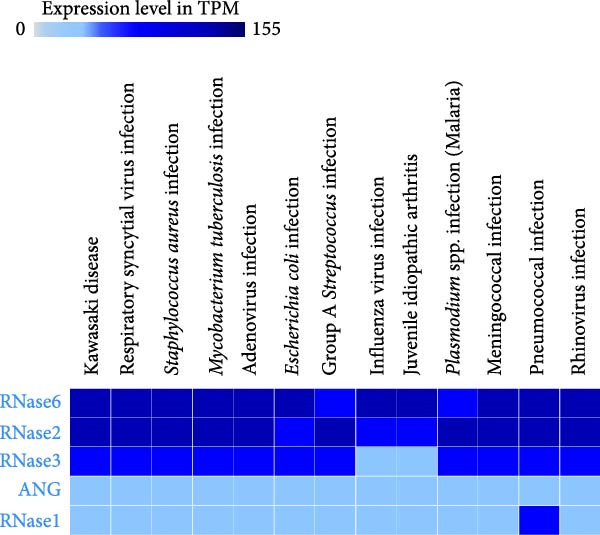
Basal RNA expression levels in whole blood from infectious diseases patients. Taken from data reported by the European Diagnostic Transcriptomic Library (EDTL) study, deposited at the EMBL‐EBI Expression Atlas.

Despite the lack of systematic comparative experimental data in the protein expression Atlas, we can identify a repetitive pattern for human RNase type overexpressed in most infection conditions, as was reported by the Diamonds European Diagnostic Transcriptomic Library (EDTL) study from whole blood RNA expression Data, which covered a wide range of conditions from bacterial, viral and parasite infections (Figure 5). Unfortunately, the data are restricted to blood cells expression pattern, and no equivalent screening assay is available for infection in mice at the expression databank, making it too premature to make conclusive remarks.

## 3. Conclusions and Future Perspectives

Nonetheless, overlapping and distinct properties of mice and human RNases should be considered before engaging studies in experimental animal models. Whereas some human RNases have a clear orthologous counterpart in mice (i.e., RNases 1, 4 and 6), others, such as EARs and Angs, show a significant gene expansion in rodents (Figure 2), hindering a straightforward analysis. Even for RNases 1, 4 and 6, their tissue distribution differs between humans and mice, adding further complexity when attempting side‐by‐side comparisons [28, 30, 31, 45, 104–107, 140, 142]. Another factor to take into consideration is the fact that tissue expression profile has been thoroughly studied in humans, but much less information is available for mice. Overall, we do observe some biological properties linked to specific family subtypes (Figure 4), such as conserved roles in both humans and mice for RNase 1 as a cardioprotective degrader of circulating extracellular RNA and for RNase 6 as a protector of urinary tract against pathogenic bacteria. Regarding RNase 4, its role in maintaining gut microbiota homeostasis in mice is well established. However, despite diminished RNase 4 levels in IBD patients, further analysis of human samples is necessary to characterise the role of this protein in controlling human gut commensal populations. Similarly, a relationship between diabetes, diminished RNase 4 levels in the urinary tract and higher risk of UTI has been established through experiments in mouse models and human samples, though further studies are required to determine the actual role of RNase 4 in host defence. Regarding RNase 7 and RNase 8, despite their exclusive presence in primates, the generation of transgenic mice containing the human *RNASE7* gene opens the possibility to obtain paramount information about its potential in vivo role in humans [153].

Generation of mice knockouts for RNases genes can also be highly informative, though their usefulness may be sometimes limited in the cases where RNases can have functional redundancy such as mEARs and mAngs [31, 105, 130, 144]. Substitution of a mouse RNase gene by its human counterpart can also be useful to study human RNase functions and to reveal divergent properties between human and mouse RNases [146]. Ultimately, there is a need to quantify mouse and human RNase levels in tissues and body fluids, as well as within distinct cellular compartments, along with their fluctuations with physiologic and pathologic states. Such studies should also take into consideration the presence of the RNase inhibitor (RNH1). In this context, when analysing the in vitro results of recombinant proteins added to cellular culture we should bear in mind the structural differences in RNH1 among vertebrates, which can significantly influence their inhibitory ability towards their RNases binding partners [186, 187]. Interestingly, a close inspection of structural properties of mouse and human RNH1 highlighted point substitutions in interface residues and surface solvated Cys residues, which account for minor differences in inhibitor relative affinities and sensitivity to oxidation (three out of four conserved surface exposed Cys in mouse vs human) [186]. Thus, hRNase–RNH1 interactions would not be fully reproduced in transgenic mouse models. These structural differences that have been originated through parallel co‐evolution between RNases and their inhibitors, might also be encountered for other RNase putative binding proteins that mediate host immunity. Overall, pros and cons of the use of experimental mouse infection models are summarised in Table 2.

**Table 2:** Summary of advantages and disadvantages of using mouse models to study the role of human RNases in host defence.

Advantages	Disadvantages
• Conserved properties in almost all RNases (digestive, antimicrobial, angiogenesis and immunomodulatory among others) • Similar expression patterns in body tissues and cell types • Conserved secretory pathways • Possibility to generate transgenic strains through diverse strategies • Possibility to faithfully reproduce most human infectious diseases • Possibility to use engineered humanised mice to evaluate specific receptor‐related roles	• Genetic expansion and fast evolutionary divergence of RNase A superfamily in rodents. • Probable functional redundancy or poorly understood specialisation in mouse EARs and ANGs subfamilies • Apparent differences in physiological function of eosinophils in humans and mice, with diminished secretion in mice • Human RNases 2 and 3 have diverged from a common ancestor during primate evolution and therefore share their mouse counterparts • Absence of mouse counterparts of human RNase 7 and RNase 8. • Slight structural differences identified in human/mouse RNH1 inhibitors • Potential significant differences in other human and mouse RNase binding partners • Differences in pathogen‐associated recognition TLRs in humans and rodents

Some of the annotated disadvantages of using mouse models might be overcome by other strategies, such as the use of human induced pluripotent stem cells (iPSCs), which can be differentiated into selected lineages and might be subjected to gene edition upon request. Nonetheless, the use of the reported strategies based on mouse models together with novel emerging technologies is expected to increase our current knowledge of RNases and serve as a base for the development of novel antimicrobial therapeutics.

## Conflicts of Interest

The authors declare no conflicts of interest.

## Author Contributions

Ester Boix and Raúl Anguita ideated the project. Raúl Anguita gathered the information, prepared figures and tables and wrote the initial draft manuscript. Raúl Anguita, Brian Becknell, Yusuf Ali and Ester Boix wrote and edited the manuscript. All contributed to the editing and revision of the text and approved the final manuscript version.

## Funding

This work was supported by the Spanish Agencia Estatal de Investigación for funding (PID2022‐137872NB‐I00). RA was the recipient of a FI‐SDUR PhD fellowship (AGAUR, Generalitat de Catalunya).

## Data Availability

Data sharing not applicable to this article as no datasets were generated or analysed during the current study.
